# One out of three bystanders of out-of-hospital cardiac arrests shows signs of pathological psychological processing weeks after the incident - results from structured telephone interviews

**DOI:** 10.1186/s13049-021-00945-8

**Published:** 2021-09-08

**Authors:** Peter Brinkrolf, Bibiana Metelmann, Camilla Metelmann, Mina Baumgarten, Carolin Scharte, Alexander Zarbock, Klaus Hahnenkamp, Andreas Bohn

**Affiliations:** 1 Department of Anaesthesiology, University Medicine Greifswald, Klinik für Anästhesiologie, Ferdinand-Sauerbruch Straße 1, 17489 Greifswald, Germany; 2 University Medicine Greifswald, Greifswald, Germany; 3 Department of Anaesthesiology, Intensive Care and Pain Medicine, University Hospital Münster, Münster, Germany; 4 City of Münster Fire Department, Münster, Germany

**Keywords:** Bystander CPR, Out-of-hospital CPR, Out-of-hospital cardiac arrest, Witnessed cardiac arrest, Psychological distress, Emotional adjustment, Psychological trauma

## Abstract

**Background:**

Witnessing an out-of-hospital cardiac arrest (OHCA) is a traumatic experience. This study analyses bystanders` psychological processing of OHCA. We examined the potential impact of bystanders performing resuscitation and the influence of the relationship between bystander and patient (stranger vs. family/friend of the patient) on the psychological processing.

**Methods:**

A telephone interview survey with bystanders, who witnessed an OHCA of an adult patient was performed weeks after the event between December 2014 and April 2016. The semi-standardized questionnaire contained a question regarding the paramount emotion at the time of the interview. In a post-hoc analysis statements given in response were rated by independent researchers into the categories “signs of pathological psychological processing”, “physiological psychological processing” and “no signs of psychological distress due to the OHCA”.

**Results:**

In this analysis 89 telephone interviews were included. In 27 cases (30.3%) signs of pathological psychological processing could be detected. Bystanders performing resuscitation had a higher rate of “no signs of psychological distress after witnessing OHCA” compared to those not resuscitating (54.7% vs. 26.7%, p < 0.05; relative risk 2.01; 95%CI 1.08, 3.89). No statistical significant differences in the psychological processing could be shown for gender, age, relationship to the patient, current employment in the health sector, location of cardiac arrest or number of additional bystanders.

**Conclusions:**

One out of three bystanders of OHCA suffers signs of pathological psychological processing. This was independent of bystander´s age, gender and relationship to the patient. Performing resuscitation seems to help coping with witnessing OHCA.

**Supplementary Information:**

The online version contains supplementary material available at 10.1186/s13049-021-00945-8.

## Background

Out-of-hospital cardiac arrest (OHCA) occurs in Europe with an incidence of 37–55 per 100,000 per year [1]. Patients surviving a cardiac arrest have a high probability of developing posttraumatic stress disorder [2, 3]. Less is known about the emotional consequences for bystanders witnessing OHCA [4]. The European Resuscitation Council Guidelines 2021 encourage research on this aspect [5].In (nearly) all cases of OHCA the emergency service is contacted by a bystander in the patient´s surrounding. In around 50% of OHCA the collapse is witnessed [6]. This is often perceived as a defining moment in life [4, 7]. Performing resuscitation is described as emotionally challenging for lay rescuers [5]. The suddenness of most OHCA, especially when leading to unexpected death and subsequent grief among the patient´s family and friends, is a traumatic experience [8, 9]. Grief is a risk factor for physical and mental ill health [10].

Paramedics recognize supporting grieving relatives after resuscitation as an important and demanding task [11]. While paramedics strive to help with the grieving process, a city of Oslo case study shows they are not taught how to do so and feel challenged by time constraints [11].

It is controversially discussed whether the risk for post-traumatic stress disorder in relatives is increased or decreased, when they are present during emergency team-led cardiopulmonary resuscitation (CPR) [12–16]. Mathiesen and colleagues interviewed 20 bystanders, who performed CPR, on how they processed this event and found that some struggled in life (feelings of guilt, reduced work capacity, weight loss, flashbacks and nightmares) even years after the event [17]. Flashback are classified as involuntary and uncontrollable reexperiencing of memories of the traumatic event, accompanied by strong sensory impressions, and a sense of ‘‘nowness’’ or of the event occurring in the present [18]. However, previous research did not systematically evaluate the impact of performing CPR vs. passively observing OHCA on psychological processing. It is not known either, whether the relationship between bystander and patient (stranger vs. family/friend) influences the psychological processing. This post-hoc analysis of data gathered in a larger study aims to analyse the bystanders´ psychological processing of OHCA some weeks after the event. Primarily, the potential impact of the bystander performing CPR or passively observing the situation and secondly, the influence of the relationship between bystander and patient (stranger vs. family/ friend of the patient) on the psychological processing is examined.

## Methods

. The data presented in this post-hoc analysis are part of a larger data-set focussing on bystanders’ perceptions after witnessing cardiac arrest. Other findings from the dataset have already been published [19].

Between December 2014 and April 2016 bystanders, who witnessed OHCA in the city of Münster (Germany), were included in the interview study. Excluded were cases, if OHCA occurred in a medical facility (e.g. doctor`s surgery, nursing home) or after the arrival of the emergency personnel. In both cases the bystanders would be medical trained individuals in their professional surrounding and might therefore have different coping strategies. Cases were also excluded, if the patient was younger than 18 years old or no CPR was attempted by the emergency personnel, because psychological impact might be different. Cases were excluded, if no bystander could be traced. All bystanders were asked to take part in a telephone interview one to seven weeks after the event. Participants were recruited by the emergency physician on duty, who ascertained the first bystander at the scene obtaining verbal consent for passing on contact data to the researchers. Due to the time-sensitive situation and the personnels’ focus on the patient, the first consent to passing on contact data to the researchers was a verbal consent (no written consent) obtained by the EMS personnel. Because we are aware, that this was a period of extreme stress for bystanders, each interview started with the question, whether the bystander still agreed to take part in the survey.

Secondary exclusion criteria resulted from missing or incomplete contact data of the bystander, lack of bystanders’ consent or failure to reach the bystander via phone for the interview.

In a third step cases were excluded, if the interview was conducted within a week of the event. We decided to exclude these cases from the analysis to minimize a potential bias by transient physiological stress reactions, as emotions and psychological processing can be expected to change and evolve within the first days. Interviews with missing response to the question concerning the paramount emotion were also excluded.

The telephone interview followed a semi-standardized questionnaire consisting of 116 items and encompassed a spectrum of different topics such as the location of the incidence, the relationship between bystander and patient and individual characteristics of the bystander such as gender, age, highest qualification/degree, as well as the paramount emotion at the time of the interview. Every item was explored with one primary open question, followed by one secondary specific question to secure full evaluation. Responses were documented verbatim.

Outcome of the CPR (survival vs. death) remained unknown to the researchers to eliminate emotional priming in the interpretation of the answers. The researchers did not know, whether the interviewee knew about the outcome of the patient at the time of the interview.

To minimize variation, a single researcher conducted all telephone interviews (author: CS). The statements of the open question of the paramount emotion at the time of the interview were independently grouped across the entire participant cohort by two researchers—an emergency physician and a specialist psychiatrist (author: BM and MB). All cases of diverging classification of responses were discussed together with two further researchers (author: CM and PB).

This article focuses on the quantitative analysis of different factors, which might influence the thoughts and emotions of bystanders at the time of interview with emphasis on the impact of performing CPR and the relationship between bystander and patient.

Chi-square test was used to assess significance levels. A relative risk with 95% confidence interval was calculated for the group of “no signs of psychological distress due to the OHCA”. Pearson’s Chi-square test and phi coefficient were used to compare telephone-CPR and bystander-CPR. A p value < 0.05 was rated significant. Statistical processing of the data was performed using Microsoft Excel 2010 (Microsoft Corporation, Redmond, Washington, USA) and IBM SPSS Statistics, version 24.0 (IBM Corporation, Armonk, New York, USA).

## Results

### Demographics of participant cohort

**Fig. 1:**
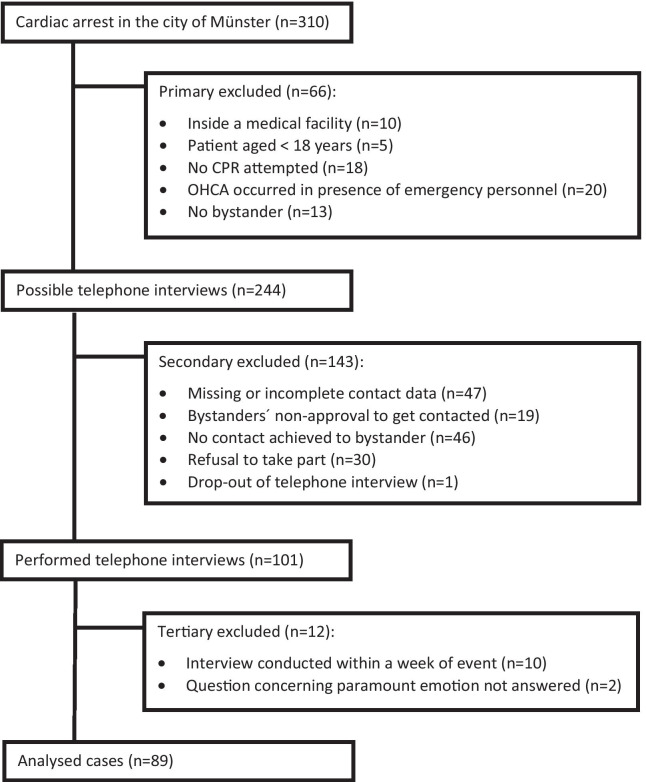
Included and excluded cases

During the study period 310 OHCA were recorded in the German Resuscitation Registry as having occurred in the city of Münster. A total of 66 cases were excluded: 10 cases occurred inside a medical facility (e.g. doctor`s surgery, nursing home), in 5 cases the patient was under 18 years old, in 18 cases resuscitation was neither attempted by bystander nor emergency team (e.g. palliative scenario), in 20 cases the ambulance team had been present when the OHCA occurred, in 13 cases no bystander was present. Out of the remaining 244 possible telephone interviews 101 (41.4%) could be conducted. In 10 cases the interview was conducted within a week from the incident. In 2 cases the question of the paramount emotion at the time of the interview was not answered. Therefore, we excluded these cases (Fig. 1).

Interview duration differed between 6 and 69 min (median 18 min). A total of 54 women and 35 men were interviewed and included in the analysis. 58 bystanders performed CPR, while 31 did not. The median age of the bystanders was 54 years. The median time between cardiac arrest and interview was 18 days (minimum 7 days, maximum 47 days). In 54 cases the bystanders were relatives (35 spouses/ partners, 16 children, 3 other relatives) of the OHCA patient, in 4 cases friends and in 6 cases colleagues. 25 bystanders did not know the cardiac arrest patient.

### Categories of psychological reaction

The bystanders’ main thoughts and principal emotion a few weeks after witnessing OHCA were grouped into four categories: “signs of pathological psychological processing” (answers such as “flashbacks”, “thin-skinned”, “jumpy”, “feeling of guilt”), “physiological psychological processing” (answers such as “affected”, “very sad, but father was very ill”), “no signs of psychological distress due to the OHCA” (answers such as “content”, “getting along”), and “not distinctly appraisable” (ambiguous phrasing; answers couldn´t be allocated to the aforementioned groups), compare Additional file 1.

**Fig. 2:**
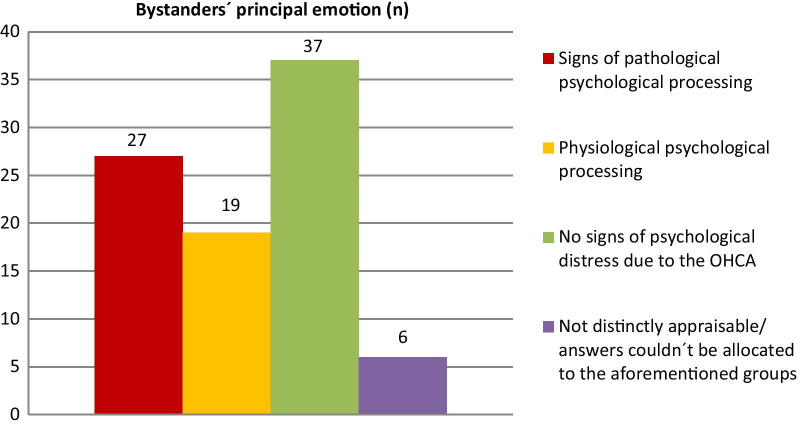
Bystanders’ principal emotion

In 27 out of 89 cases signs of pathological psychological processing could be detected. In 19 out of the 89 cases the main feeling of the bystander could be qualified as a physiological psychological processing, while 37 bystanders showed no signs of psychological distress due to the experience. 6 cases were not distinctly appraisable, as answers couldn´t be allocated to the aforementioned groups (Fig. 2).

In 73 (82.1%) cases the bystander’s principal emotion were categorized the same by the two independent raters. In 16 cases (17.9%) the principal emotion was rated differently and the category was determined together with a third and fourth rater.

Table 1 documents individual characteristics, such as age, gender and relationship with patient and circumstances of the event such as location, number of additional bystanders.

**Table 1:** Bystanders’ principal emotion grouped after different individual-related and situational factors

	Total number	Signs of pathological psychological processing (%)	Physiological psychological processing (%)	No signs of psychological distress due to the OHCA (%)
All bystanders	83	27 (32.5%)	19 (22.9%)	37 (44.6%)
*Gender (p = 0.1523)*				
Male	33	8 (24.2%)	6 (18.2%)	19 (57.6%)
Female	50	19 (38%)	13 (26%)	18 (36%)
*Age (p = 0.2372)*				
< 35 years	12	2 (16.7%)	3 (25%)	7 (58.3%)
35–64 years	52	18 (34.6%)	9 (17.3%)	25 (48.1%)
> 64 years	19	7 (36.85%)	7 (36.85%)	5 (26.3%)
*Professional or voluntary work in the health sector (p = 0.1183)*				
Yes	18	7 (38.9%)	1 (5.6%)	10 (55.5%)
No	62	19 (30.7%)	18 (29%)	25 (40.3%)
*Relationship between patient and bystander (p = 0.3369)*				
Know each other	60	22 (36.7%)	14 (23.3%)	24 (40%)
Don´t know each other	23	5 (21.75%)	5 (21.75%)	13 (56.5%)
*Degree of family (p = 0.4812)*				
Spouse/ partner of patient	31	10 (32.25%)	11 (35.5%)	10 (32.25%)
Child of patient	16	6 (37.5%)	3 (18.7%)	7 (43.8%)
*Location of cardiac arrest (p = 0.5059)*				
Home	57	17 (29.8%)	15 (26.3%)	25 (43.9%)
At work or in public	26	10 (38.5%)	4 (15.4%)	12 (46.1%)
*Number of additional bystanders (p = 0.3657)*				
0	35	14 (40%)	10 (28.6%)	11 (31.4%)
1	18	5 (27.8%)	3 (16.7%)	10 (55.5%)
More than 1	30	8 (26.7%)	6 (20%)	16 (53.3%)
*Telephone-CPR by dispatcher (p < 0.05)*				
Yes	30	6 (20%)	11 (36.7%)	13 (43.3%)
No	52	21 (40.4%)	8 (15.4%)	23 (44.2%)
*Bystander performed CPR (p < 0.05)*				
Yes	53	14 (26.4%)	10 (18.9%)	29 (54.7%)
No	30	13 (43.3%)	9 (30%)	8 (26.7%)
*Witnessed a cardiac arrest before (p = 0.8931)*				
Yes	22	8 (36.4%)	5 (22.7%)	9 (40.9%)
No	61	19 (31.1%)	14 (23%)	28 (45.9%)
*Person was responsive at the beginning (p = 0.6503)*				
Yes	17	6 (35.3%)	5 (29.4%)	6 (35.3%)
No	66	21 (31.8%)	14 (21.2%)	31 (47%)
*Initial pattern of breathing (p = 0.6844)*				
No breathing	36	10 (27.8%)	8 (22.2%)	18 (50%)
Agonal breathing	28	11 (39.3%)	6 (21.4%)	11 (39.3%)
Breathing not remembered	14	4 (28.6%)	5 (35.7%)	5 (35.7%)
*Bystander was assured by something (e.g. bystanders, experiences) (p = 0.4621)*				
Yes	48	13 (27.1%)	12 (25%)	23 (47.9%)
No	35	14 (40%)	7 (20%)	14 (40%)
*First thought (p = 0.5445)*				
I knew what to do	37	14 (37.8%)	5 (13.5%)	18 (48.7%)
I did not know what to do	12	3 (25%)	4 (33.3%)	5 (41.7%)
I was terrified	26	9 (34.6%)	7 (26.9%)	10 (38.5%)
First thought not remembered	7	1 (14.3%)	3 (42.85%)	3 (42.85%)

The six cases grouped as “not distinctly appraisable” were not included into the significance testing.

**Fig. 3:**
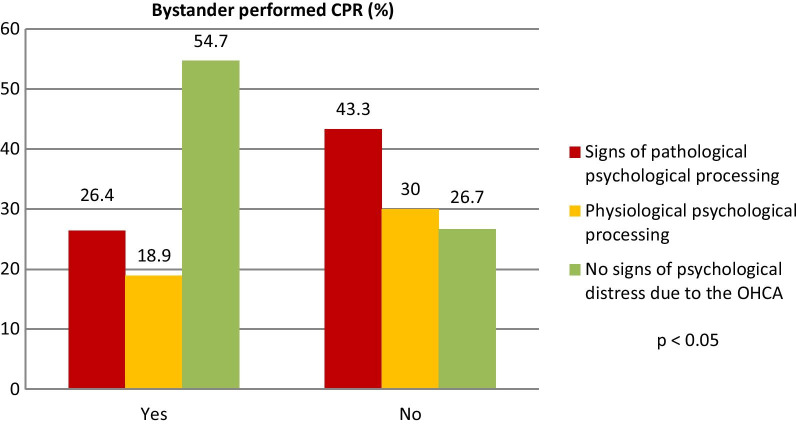
Probability of different psychological processing of the bystander in the group of bystanders performing CPR or not. No signs of psychological distress due to OHCA are significantly more often in cases, where the bystander performed CPR

Bystanders performing resuscitation had a significant higher rate of “no signs of psychological distress after witnessing OHCA” compared to those not resuscitating (54.7% vs. 26.7%, p < 0.05; relative risk 2.01; 95%CI 1.08, 3.89), see Fig. 3.

**Fig. 4:**
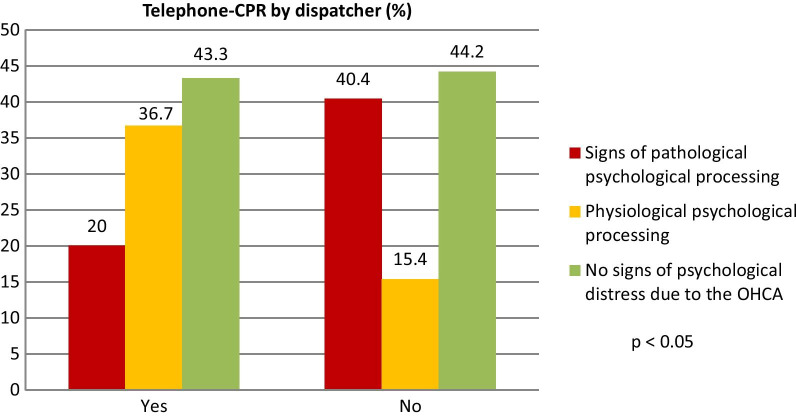
Probability of different psychological processing of the bystander in cases with and without dispatcher-instructed CPR in percentage. Signs of pathological psychological processing were significantly less in cases of telephone-CPR

In cases, where the dispatcher explained and instructed CPR, signs of pathological psychological processing were significantly less compared to cases without telephone-CPR (20% vs. 40.4%, p < 0.05), see Fig. 4. However, the relative risk for no signs of psychological distress didn´t significantly differ if telephone-CPR was performed or not (0.98; 95%CI 0.59, 1.63).

There were no significant differences in the psychological processing depending on individual-related factors such as gender, age, relationship to the patient and work in the health sector or situational factors such as location of cardiac arrest and number of additional bystanders.

Pearson’s Chi-square test showed a significant correlation of medium strength between telephone-CPR by dispatcher and bystander performed CPR (φ = 0.28; p = 0.008). In two cases it could not be determined, whether the dispatcher guided CPR, those were not included in the analysis.

## Discussion

This post-hoc analysis presents findings on bystanders´ psychological processing of OHCA. It focusses on the potential impact of the bystander performing CPR and the influence of the relationship between bystander and patient.

As the new European Resuscitation Council Guidelines point out in the Ethics chapter, more studies are needed on debriefing witnesses of cardiac arrest [5]. In this article we introduce the hypothesis that every third bystander of out-of-hospital cardiac arrest shows signs of pathological psychological processing weeks after the incident. Furthermore we indicate that performing bystander-CPR might increase the likelihood of dealing with the experience without developing signs of psychological distress.

Previous studies showed, that families of patients with acute syndromes (e.g. myocardial infarction) show psychological distress [7, 20–23]. Witnessing an ambulance team performing CPR on relatives is associated with an increased risk of depression 90 days after the event [24]. Interestingly, our analysis provides indication that this might also be true, if patient and bystander did not know each other before the event. More than every fifth bystander, who witnesses the cardiac arrest of a stranger, shows signs of pathological psychological processing. Even though the bystander is not acquainted with the person, he has problems coping with the processing of the experience. Because the person is neither a family member nor a friend, it does not fit into the culturally accepted pattern of grieving a bereavement.

Signs of pathological psychological processing might occur only a short time but might also continue over longer periods influencing the life of this person in multiple aspects [7]. In a study by Van't Wout Hofland one out of three caregivers of survivors after cardiac arrest experienced high level of trauma related stress even two years after the event. This was intensified, if caregivers witnessed the cardiac arrest [25]. It is therefore important to understand, which persons show signs of pathological psychological processing and identify factors, that might enhance the risk [7].

Our findings point to lower rates of pathological psychological processing in cases, where the dispatcher guided the bystander via telephone-CPR. Clear instructions on what to do are highly appreciated by the bystanders [26] and might be perceived as easing the responsibility load by sharing with the professional on the telephone line. Since structured emotional and psychological support for relatives witnessing in-hospital resuscitation can decrease the likelihood of psychological disorders [15], telephone guidance by dispatchers might be beneficial as well. Telephone guidance might help a bystander to gain some control of the situation. Perceived control of a situation is associated with lower distress level and less posttraumatic stress disorder symptoms [27, 28]. This might reassure the bystander and therefore help him to process this experience. Out of the 48 bystanders, who reported feeling assured by some factors in the situation, 13 mentioned receiving telephone-guidance.

However, feeling assured by some factors only resulted in a trend towards fewer signs of pathological psychological processing that did not reach significance.

The parameters “dispatcher explained telephone-CPR” and “bystander performing CPR” are correlated. Concordantly, a significant lower rate of pathological psychological processing occurred in cases where the bystander performed CPR. Persons trusting that their actions have the power to positively influence their relative´s health problems experience less anxiety and depression [29]. Performing resuscitation is hard physical work, which leads to severe exhaustion of the bystander. This might help the bystander to remember, that he did everything he could do to save the person from cardiac arrest.

The active participation in the treatment of the patient might lead to a feeling of empowerment. However, in contrast to our study Van't Wout Hofland found no differences in the level of impact of event for caregivers, who witnessed a cardiac arrest and performed CPR in comparison to those, who did not perform CPR [25].

Although there is a trend towards more signs of pathological psychological processing among women than men, it did not reach significance. Likewise, there was no significant difference in signs of pathological psychological processing between the age groups of the bystander < 35 years vs. 35–64 years vs. > 65 years.

To avoid the complications of pathological psychological processing in bystanders the authorities of emergency systems should plan, develop and implement measures to support bystanders through information on available support and easy access to professional assessment and help where needed [4]. Mausz and Tavares contacted 15 bystanders of cardiac arrest two weeks after the incident and performed a structured interview [30]. Bystanders had difficulties dealing with the distressing images and participants appreciated the opportunity of a debriefing with a health care professional [30]. Based on this a structured debriefing pathway was developed [31]. This could be a starting point to develop a support system, that applies to families of OHCA victims as well as to strangers, who witnessed OHCA. In our study, we did not determine, if and how the EMS providers debriefed the bystanders. At the time of the study, structured debriefing hadn´t been implemented in local OHCA-protocols. It is likely, that debriefing has a major influence on bystanders´ coping. Hence, this should be part of future research [5].

We see an important additional benefit of campaigns promoting bystander- cardiopulmonary resuscitation [32–34]. While bystander-CPR enhances the probability of surviving a cardiac arrest [35–38], our analysis also indicates that it can increase the likelihood of bystanders not developing signs of distress in consequence of the event.

### Limitations

The general transferability of this study to other regions is reduced by the single-centre-design.

The study protocol included a wide range of different aspects regarding bystanders of OHCA and was initially developed for analysing a different research question. This paper is a post-hoc analysis. Because the telephone interview addressed multiple research questions, a systematic psychological testing of the bystanders was not undertaken. The individual answers of the OHCA witnesses were later grouped into four categories. These categories were not based on a validated instrument. Although this process was done by four authors, the grouping could lead to a bias, because answers could be ambiguous or unclear. A translation of all answers is attached as Additional file 1.

Psychological processing is complex and changes over the time and emotions and mental state are expected to evolve and differ between first days, weeks and months. Conducting the interview at a different time point might have led to different results.

We do not know, whether the patients survived the cardiac arrest. Therefore, we cannot determine the impact of survival on the feelings of the bystander. Then again, if the bystander was a stranger to the patient, details regarding survival of the patient will have remained unknown. No significant differences in the coping between bystanders who did or did not know the person were shown.

The response rate was 41.4%, which might imply a considerable non-responder-bias. A total of 143 bystanders could not be interviewed. However, of those 93 bystanders could repeatedly not be reached. Only 31 bystanders actively refused to participate in the study.

## Conclusions

Our data give reason for concern, that one out of three bystanders of OHCA suffers signs of pathological psychological processing. The probability of such reactions was reduced in cases, where the dispatcher explained telephone-CPR and in cases, where the bystander started CPR. Interestingly, more than every fifth bystander, witnessing the cardiac arrest of a stranger, showed signs of pathological psychological processing. Our findings point to potential approaches for emergency authorities, including implementation of assessment of and support for bystanders, who witnessed cardiac arrest and campaigns promoting bystander-CPR. Further studies including an assessment of psychological and psychiatric signs and symptoms in bystanders are needed.

## Supplementary Information

**Additional file 1**: Translation of all answers, grouped into the four categories.

## Data Availability

The datasets used and analysed during the current study are available from the corresponding author on reasonable request.

## Abbreviations

OHCA: Out-of-hospital cardiac arrest
CPR: Cardiopulmonary resuscitation
