# Supplementary material for: One out of three bystanders of out-of-hospital cardiac arrests shows signs of pathological psychological processing weeks after the incident - results from structured telephone interviews
Source: Scand J Trauma Resusc Emerg Med. 2021 Sep 8;29:131. doi: 10.1186/s13049-021-00945-8 (PMC8425096; doi:10.1186/s13049-021-00945-8)
Supplement: Supplementary file 1 — Additional file 1: Translation of all answers, grouped into the four categories. [file 13049_2021_945_MOESM1_ESM.docx]

**Additional File 1: translation of all answers, grouped into the four categories**

| **Category** |  | **number (total n=89)** |
| --- | --- | --- |
| **Signs of pathological psychological processing (n = 27)** | | |
|  | Repeatedly reliving the situation | 19 |
|  | Thin-skinned *[verbatim answer]* | 1 |
|  | Jumpy *[verbatim answer]* | 1 |
|  | Feelings of guilt | 4 |
|  | Flashbacks | 2 |
| **Physiological psychological processing (n= 19)** | | |
|  | Affected | 10 |
|  | Very sad, but father was very ill *[verbatim answer]* | 1 |
|  | Ambivalent *[verbatim answer]* | 1 |
|  | Try to distract myself | 3 |
|  | Never want to experience it again *[verbatim answer]* | 1 |
|  | Sought family counseling *[verbatim answer]* | 1 |
|  | She should have died in peace *[verbatim answer]* | 1 |
|  | Did I do everything correct? *[verbatim answer]* | 1 |
| **No signs of psychological distress due to the OHCA (n = 37)** | | |
|  | Content | 5 |
|  | Getting along | 26 |
|  | I think little/not at all about the situation. | 1 |
|  | Lucky *[verbatim answer]* | 1 |
|  | Glad to have been there for him *[verbatim answer]* | 1 |
|  | It is better that way *[verbatim answer]* | 1 |
|  | In mourning *[verbatim answer]* | 1 |
|  | I expected it *[verbatim answer]* | 1 |
| **not distinctly appraisable/ answers couldn´t be allocated to the aforementioned groups (n= 6)** | | |
|  | CPR *[verbatim answer]* | 1 |
|  | I did everything I could but that was not enough *[verbatim answer]* | 1 |
|  | Something like that should never happen again *[verbatim answer]* | 1 |
|  | I cannot believe it *[verbatim answer]* | 1 |
|  | Got certainty *[verbatim answer]* | 1 |
|  | I had to function *[verbatim answer]* | 1 |
